# Machine Learning-Supported Enzyme Engineering toward Improved CO_2_-Fixation of Glycolyl-CoA Carboxylase

**DOI:** 10.1021/acssynbio.3c00403

**Published:** 2023-11-20

**Authors:** Daniel G. Marchal, Luca Schulz, Ingmar Schuster, Jelena Ivanovska, Nicole Paczia, Simone Prinz, Jan Zarzycki, Tobias J. Erb

**Affiliations:** † Department of Biochemistry and Synthetic Metabolism, Max-Planck-Institute for Terrestrial Microbiology, Marburg 35043, Germany; ‡ Exazyme GmbH, Berlin 13355, Germany; § Core Facility for Metabolomics and Small Molecule Mass Spectrometry, Max-Planck-Institute for Terrestrial Microbiology, Marburg 35043, Germany; ∥ Central Electron Microscopy Facility, Max-Planck-Institute of Biophysics, Frankfurt 60438, Germany; ⊥ SYNMIKRO Center for Synthetic Microbiology, Marburg 35032, Germany

**Keywords:** photorespiration, CO_2_ fixation, machine learning, directed evolution, enzyme engineering, glycolyl-CoA carboxylase

## Abstract

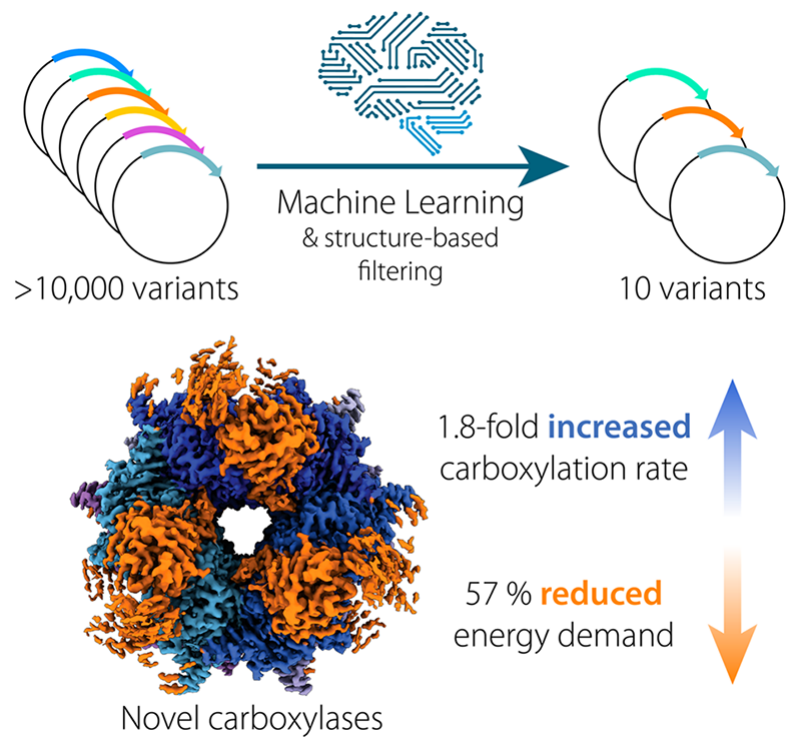

Glycolyl-CoA carboxylase (GCC) is a new-to-nature enzyme that catalyzes the key reaction in the tartronyl-CoA (TaCo) pathway, a synthetic photorespiration bypass that was recently designed to improve photosynthetic CO_2_ fixation. GCC was created from propionyl-CoA carboxylase (PCC) through five mutations. However, despite reaching activities of naturally evolved biotin-dependent carboxylases, the quintuple substitution variant GCC M5 still lags behind 4-fold in catalytic efficiency compared to its template PCC and suffers from futile ATP hydrolysis during CO_2_ fixation. To further improve upon GCC M5, we developed a machine learning-supported workflow that reduces screening efforts for identifying improved enzymes. Using this workflow, we present two novel GCC variants with 2-fold increased carboxylation rate and 60% reduced energy demand, respectively, which are able to address kinetic and thermodynamic limitations of the TaCo pathway. Our work highlights the potential of combining machine learning and directed evolution strategies to reduce screening efforts in enzyme engineering.

## Introduction

Photosynthesis plays a crucial role in the global carbon cycle by converting CO_2_ to organic compounds that feed virtually all life on Earth. However, one limiting factor in photosynthesis is the carbon conversion efficiency of the Calvin–Benson–Bassham cycle and in particular its key enzyme ribulose-1,5-bisphosphate carboxylase/oxygenase (Rubisco). Besides fixing CO_2_, Rubisco also captures O_2_ as a side reaction.^1^ This undesired reaction with O_2_ yields 2-phosphoglycolate, which needs to be recycled in a process called photorespiration, resulting in the loss of previously fixed carbon.

To circumvent the loss of carbon during photorespiration, we recently developed the tartronyl-CoA (TaCo) pathway, a synthetic carboxylation module, which additionally fixes CO_2_ during photorespiration.^2^ Theoretical and experimental data show that the TaCo pathway indeed improves carbon yield during photosynthesis.^2−4^ The key enzyme in the TaCo pathway is a new-to-nature enzyme, glycolyl-CoA carboxylase (GCC), that we established through structure-guided approaches and large-scale screening of mutagenesis libraries of propionyl-CoA carboxylase (PCC) from *Methylorubrum extorquens*.^2^ PCC is a biotin-dependent carboxylase that consists of two subunits. The α-subunit comprises a biotin carboxylase domain and a biotin-carboxyl-carrier protein (BCCP) domain. The β-subunit comprises only a carboxyl transferase domain. The enzyme forms an α_6_β_6_ dodecameric complex, where the β-subunits arrange in a central core of two trimeric layers, while the α-subunits sit on top of the core and face outward.^5^ The biotin cofactor that is essential to catalysis is covalently linked to a lysine residue in the BCCP domain of the α-subunit and acts as a flexible arm that transfers the carboxyl group derived from HCO_3_^–^ between the active sites of the α- and β-subunits.^6^ In iterative rounds, we introduced five mutations into PCC to create variant GCC M5. This quintuple substitution variant carboxylates glycolyl-CoA at a catalytic rate of 5.6 ± 0.3 s^–1^, which is comparable to that of natural biotin-dependent carboxylases.

Despite a more than 1000-fold improvement in activity, the catalytic efficiency of GCC M5 still lags about 4-fold behind that of native PCC.^2^ Additionally, the enzyme catalyzes some futile ATP hydrolysis: while for PCC the stoichiometric ratio of consumed ATP per carboxylation (ATP/CO_2_) equals 1, GCC M5 hydrolyzes about 4 ATP per carboxylation, which is likely caused by a release of CO_2_ from the carboxybiotin cofactor without a fruitful carboxylation event.^2^ For the further engineering of the enzyme, a workflow exists that builds on the testing of (randomly) generated variants of GCC M5 in plate-reader based assays.^2^ However, this setup is limited by the number of screenings that can be performed per iteration, which makes it difficult to exhaustively screen the sequence space of GCC in this workflow without additional guidance.

In the last two decades, a variety of machine learning tools have been developed that support enzyme engineering by allowing simplification of the approaches and reduction of screening efforts.^7^ Machine learning (ML) is a statistical methodology that uses algorithms to learn from data for prediction and/or decision-making. ML perceives information about the sequences and properties of enzymes, processes those, and infers novel information that likely provides improved or refined properties.^8^ These algorithms are used in synthetic biology for many applications ranging from the optimization of genetic or metabolic networks^9^ over the directed evolution of enzymes^10,11^ to the prediction of kinetic properties for uncharacterized enzymes^12^ and even the *de novo* design of whole proteins.^13^

ML-assisted enzyme engineering workflows generally comprise the generation of data sets of variants of enzymes, either collected experimentally or from a database, representation of those variants in descriptor space, assigning enzyme properties to variants, and splitting data sets into training, validation, and test subsets. The final training model of variants is subsequently used for the prediction of novel or improved enzyme properties that are then validated experimentally.^7^ For example, Madani et al. trained a transformer model on 280 million protein sequences from >19,000 families to derive a *de novo* member of the lysozyme family with a wild-type catalytic rate but a maximum sequence identity of only 40% compared to lysozymes in the training data.^14^ Similarly, Ma et al. explored the use of a random forest regressor to predict activity and guide directed evolution of an imine reductase by training with a set of experimentally characterized enzyme variants.^15^ Voutilainen et al. applied a novel machine learning model utilizing Gaussian processes and featured learning for the third mutagenesis of a 2-deoxy-d-ribose 5-phosphate aldolase leading to a strong improvement in enzyme performance.^16^

Here, we present an advanced engineering workflow for GCC that we complemented by a ML algorithm to reduce the screening effort for the directed evolution of GCC toward higher carboxylation rates and reduced ATP demand. We demonstrate the successful application of the workflow by presenting two improved GCC variants. One variant shows an almost 2-fold increase in turnover rate for the carboxylation of glycolyl-CoA, while the other variant has a more than 2-fold decreased ATP per carboxylation ratio. Cryogenic electron microscopy (cryo-EM) structures combined with additional biochemical characterizations provide insights into the role of these mutations.

## Results and Discussion

The field of protein engineering has recently witnessed a growing use of ML methods. These methods have been employed to predict protein structures and enhance enzyme properties, such as stability, function, and solubility. To facilitate the engineering of GCC M5 and other proteins, we developed and assessed ML algorithms and designed a customized two-step workflow. The first step in our approach involves the utilization of ML models to predict how protein sequence corresponds to function without relying on data regarding secondary, tertiary, or quaternary protein structures. This enables us to infer the functional properties of proteins solely based on their amino acid sequences. In the second step, our prediction algorithm is integrated within a black-box Bayesian optimization loop. This loop serves as a decision-making process to select the most promising candidates for the subsequent batch of wet lab experiments. By employing this optimization loop, we can simultaneously optimize various parameters of interest, such as stability, catalytic speed, and substrate specificity. This allows for a more efficient and comprehensive optimization process in protein engineering.

We developed our ML algorithm using reproducing kernel Hilbert space methods as a basis for a Gaussian Process (GP) regression model to predict enzymatic properties such as catalytic speed and ATP efficiency.^17^ We compared it to Unirep 1900 embeddings combined with a random forest regressor developed by Ma et al.^15^ in a cross validation scheme and selected the GP model based on the performance in terms of rank correlation between predicted and measured properties on the validation sets (GP ρ = 0.42, Unirep + random forest ρ = 0.39). The GP was subsequently used to rank candidate sequences for synthesis.

**Figure 1:**
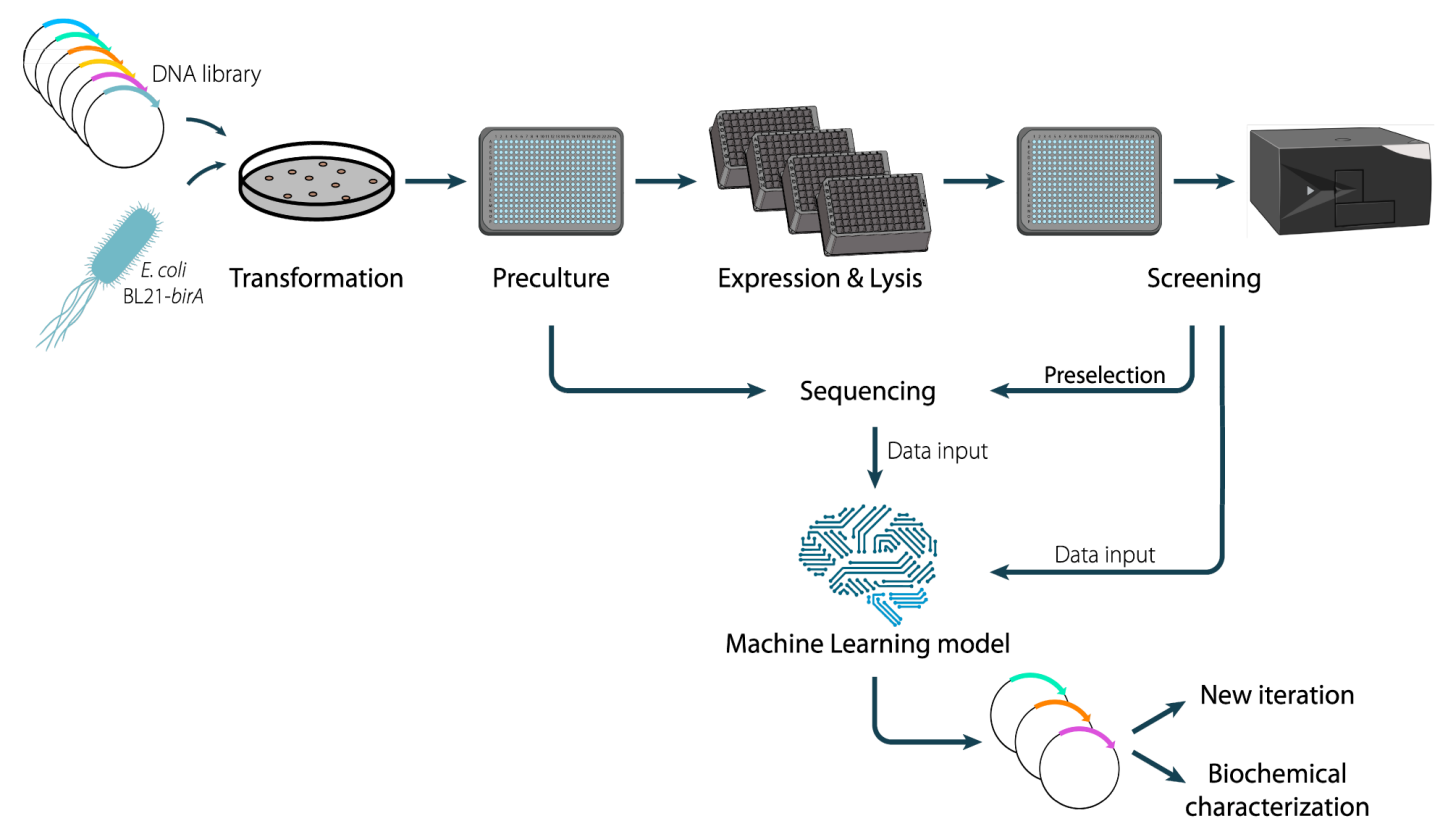
Workflow of machine learning-supported directed evolution of GCC M5. The workflow comprises the creation and transformation of randomly mutagenized GCC M5 plasmids into *E. coli* BL21-*birA*, the precultivation in 384-well plates, the protein overproduction in 96-deep well plates, chemical cell lysis, and a plate-reader based enzyme activity assay to determine kinetic properties. Based on the screening results, a subset of screened samples is selected for sequencing. Screening and sequencing data are fed to the ML model that processes the data and infers a list of promising mutations for *in vitro* testing ranked by predicted efficiency. Based on the list, the number of samples to screen can be reduced. Finally, a new iteration of the workflow can be executed, or biochemical characterization of the selected variants is started.

A data set to train the ML algorithm was prepared by using a directed evolution workflow of GCC M5 described before (Figure 1).^2^ We first created an error-prone PCR-based plasmid library of randomly mutagenized GCC M5. For this library, we neglected the PccA subunit, which is involved in the ATP-dependent carboxylation of biotin and has not been mutagenized before. We instead focused the library exclusively on the PccB subunit that interacts with glycolyl-CoA and catalyzes the release of CO_2_ from carboxybiotin and its actual transfer onto the substrate.

We performed lysate-based enzyme screens for over 3000 variants but did not observe a significant improvement of either the carboxylation rate or the ATP per carboxylation ratio (Figure S1 in the Supporting Information). From the obtained data, a subset of 161 representative candidates covering the range from inactive to most active candidates was sequenced and used to train the ML model for the prediction of beneficial enzyme variants (Supplementary file 1).

The two ML models (a GP-based model comparing positions and a Unirep-random forest model) were evaluated in a 10-fold cross validation scheme (90% train, 10% test splits) on the data set of 161 unique enzyme sequences with measured carboxylation rates and ATP per carboxylation ratios. The GP based model showed higher rank correlation (Spearmans ρ = 0.42) between the upper confidence bound of the GP and the measurements and was thus chosen for subsequent *in silico* evaluation of mutants (Figure S2). The upper confidence bound is a principled approach to black-box optimization based on GP regression models. We generated all single mutations based on GCC M5 and sorted them by the upper confidence bound criterion calculated from the GP regression model to obtain a list with ranked mutations.

The ML algorithm returned a list of all possible single mutations in the β-subunit (i.e., PccB) of GCC M5 ranked by their predicted efficiency. This list was used as a template for further *in silico* investigation and assessment of candidates that in our view might show promise for improved kinetic properties. From the top 1% (covering 105 predictions; Supplementary file 2), we created homology models based on PDB 6YBQ(2) and further investigated the structure of these variants. Variants with substitutions in the His-tag were excluded as well as substitutions that were likely to cause major steric clashes. We further considered variants which we assumed to have an impact on enzymatic activity and/or whose substitution sites had a high frequency (i.e., were presented multiple times) in the top 1% predictions. Based on these considerations, we selected seven variants to be tested *in vitro*. Additionally, we selected three variants that were ranked in the top 5% of predictions and whose positions had already been targeted during the development of GCC M5 in the past. Table S4 in the Supporting Information lists the ten candidate enzymes that were selected for biochemical characterization *in vitro*.

**Figure 2:**
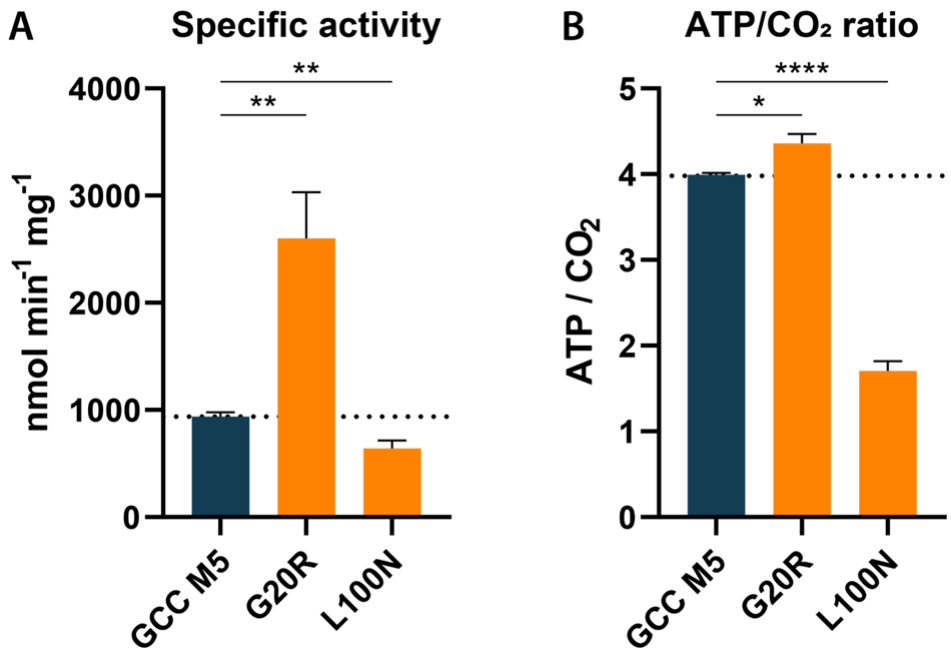
Spectrophotometric measurements of GCC M5 variants G20R and L100N. (A) Specific activities for glycolyl-CoA carboxylation were determined photometrically in a coupled enzyme assay with CaMCR and 0.5 mM glycolyl-CoA. (B) ATP per carboxylation ratios were determined by measuring specific activities for glycolyl-CoA carboxylation under ATP-limited conditions. Bars represent mean ± SD, *n* = 3–6. For significance analysis, an unpaired *t* test was performed. ****p < 0.0001, **p < 0.007, *p = 0.0216.

In the next step, we used our workflow, to screen the ten candidates in cell lysate for activity (Figure S3). In this screen, all candidates except variant M64R were still active, corresponding to a fraction of active variants of 90%. This 90% positive hit rate already marked a dramatic improvement to our prior screening efforts of random mutagenesis libraries, in which only less than 20% of variants showed measurable activities.^2^ Thus, the enrichment of active variants based on ML and manual filtering proved the benefit of combining conventional screening methods with computational tools. To quantify carboxylation rates and the ATP per carboxylation stoichiometry, we purified the remaining nine active variants and measured their activities using a spectrophotometric assay (Table S6). Among the tested candidates, variant G20R stood out with a specific activity for glycolyl-CoA carboxylation of 2.6 ± 0.4 μmol min^–1^ mg^–1^ at a concentration of 0.5 mM glycolyl-CoA, which corresponded to a 2.8-fold improvement of the carboxylation rate compared to that of GCC M5 (Figure 2, Table S6). We then performed more in detail biochemical characterizations determining *V*_max_, *k*_cat_, and *K*_M_ values for this enzyme variant using LC-MS assays, which underscored the catalytic improvement over GCC M5 (Table 1). While the apparent *K*_M_ value for glycolyl-CoA slightly increased, the *V*_max_ was 1.8-fold higher than that of GCC M5 and thus the G20R variant with a *k*_cat_ of 9.8 ± 0.2 represents a very promising candidate for GCC-based applications. In this variant, the substitution of Gly by Arg on a surface loop of the β-core strongly increased the carboxylation rate, whereas the ATP per carboxylation ratio changed only marginally (Figure 2B). Besides G20R, we also identified variant L100N that showed a significantly decreased ATP to carboxylation ratio of 1.7 ± 0.1 ATP, lowering the energy demand for the reaction by 60% compared to GCC M5 (4.0 ± 0.0 ATP per glycolyl-CoA carboxylation). In this variant, Leu100 in the active site periphery (second shell) was replaced by Asn. This substitution had been investigated already early on during GCC development, but only with the focus on the carboxylation rate and also not in the context of the M5 variant.^2^ Therefore, the benefit of this substitution had remained hidden then. All other tested variants showed similar catalytic properties compared to those of GCC M5, resulting in a discovery ratio of 20% of variants with improved kinetic properties. This number represents a great improvement compared to conventional directed evolution approaches without the support of ML algorithms, where in our previous experiments less than 0.1% of screened enzymes exhibited significantly improved properties. To evaluate whether other mutations at positions 20 and 100 showed beneficial impacts on the GCC’s catalytic properties, we tested site-saturation libraries for both positions and applied the lysate-based screen. However, we could not detect any other beneficial substitutions apart from G20R and L100N or the L100S variant from our earlier work.^2^ This observation underlines the reliability of the ML-model in predicting the best performing mutations.

**Table 1:** Kinetic Properties of GCC M5 Variants G20R and L100N^a^

enzyme	substrate	*V*_max_ [nmol min^–1^ mg^–1^]	*k*_cat_ [s^–1^]	app. *K*_M_ [mM]	*k*_cat_/*K*_M_ [s^–1^ M^–1^]	ref
GCC M5	glycolyl-CoA	2590 ± 130	5.6 ± 0.3	0.15 ± 0.03	3.63 × 10^4^	(2)
G20R	glycolyl-CoA	4520 ± 90	9.8 ± 0.2	0.27 ± 0.02	3.64 × 10^4^	this work
	acetyl-CoA	2250 ± 200	4.9 ± 0.4	0.38 ± 0.10	1.30 × 10^4^	this work
L100N	glycolyl-CoA	790 ± 40	1.7 ± 0.1	0.32 ± 0.05	5.36 × 10^3^	this work
	acetyl-CoA	174 ± 12	0.38 ± 0.03	0.63 ± 0.10	5.96 × 10^2^	this work

a The data represent means ± SD, as determined from *n* = 18 independent measurements using nonlinear regression.

**Figure 3:**
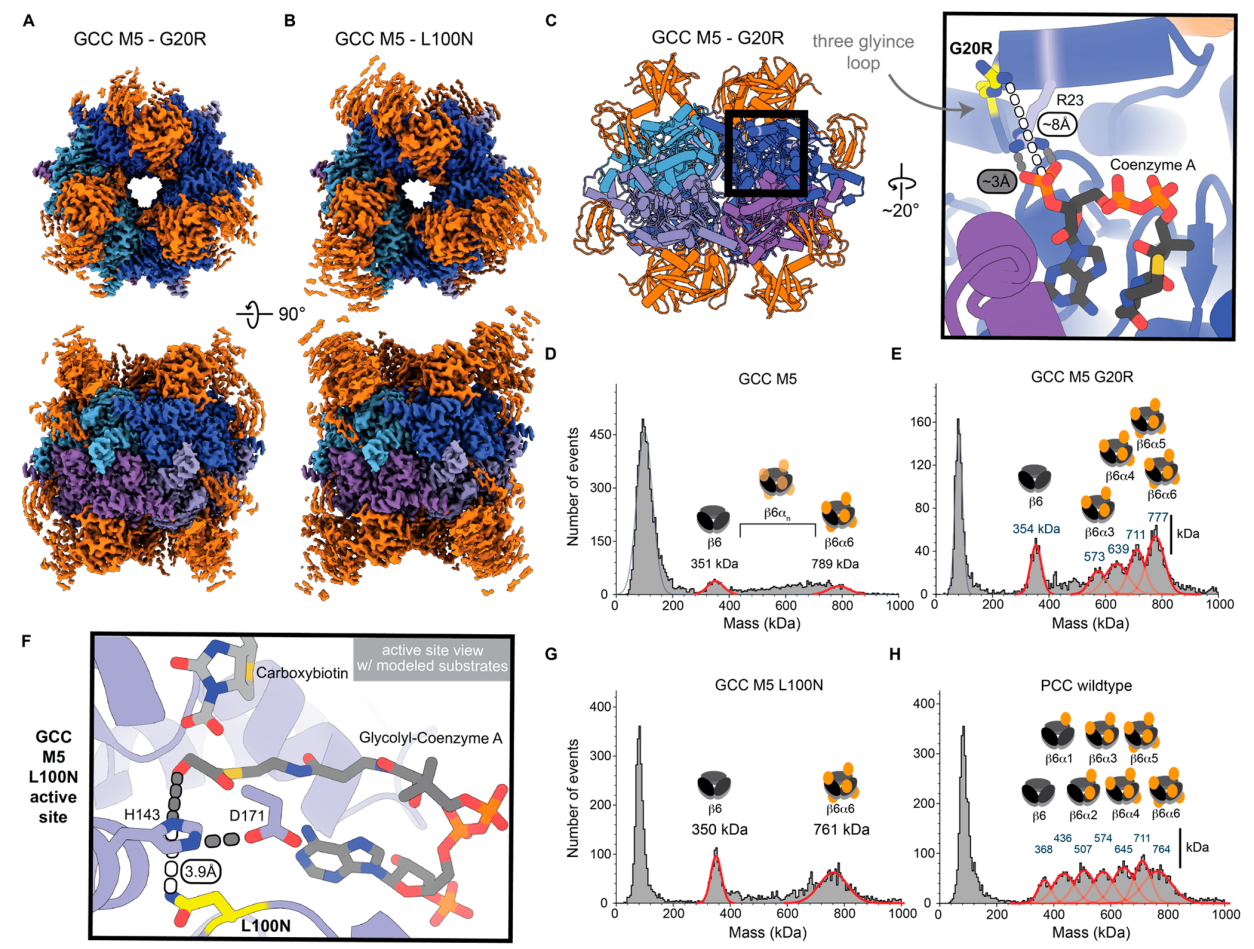
Structural analyses of the new GCC variants. (A, B) Surface representations of the cryo-EM electron density maps for the G20R (EMD-17777) and L100N (EMD-17778) variants, respectively. The β-subunits are depicted in blue tones, whereas the partial electron densities for the α-subunits are colored in orange. (C) Location of the G20R substitution (PDB 8PN7) on the surface of the β-subunit core (left panel) and a close-up showing the position of Arg20 with respect to the binding site for the adenosyl moiety of CoA (right panel). (D, E) MP experiments for the GCC M5 and G20R variants, respectively. Both variants show a wide distribution of complexes with differing numbers of α-subunits attached to the β_6_-core, highlighting a transient interaction. The G20R variant appears to favor the formation of β_6_α_6_ complexes. (F) Close-up of L100N variant active site (PDB 8PN8) showing the environment of His143 and its assumed interaction with glycolyl-CoA. Glycolyl-CoA was modeled corresponding to methylmalonyl-CoA in PDB 1ON3 with additional manual fitting that reflects the binding of CoA in the cryo-EM structures. A manually fitted carboxybiotin is shown in its most likely position for carboxyl transfer to the substrate. His143 engages in polar interactions (gray) with the glycolyl-CoA and Asp171. The amide group of L100N (yellow) is positioned parallel to the imidazole ring of His143 at a distance of 3.9 Å. (G) MP experiment for the L100N variant. L100N appears to slightly favor β_6_α_6_ complex formation in comparison to GCC M5 (panel D). (H) MP experiment for PCC from *M. extorquens* for comparison, demonstrating no clear favorability for any one oligomeric state.

To identify structural changes that might be responsible for the catalytic improvements of the G20R or L100N variants, we solved their cryo-EM structures at 2.05 and 2.31 Å, respectively (Figure 3, Figures S4 and S5). The G20R substitution is located ∼8 Å away from the 3′-phosphate of coenzyme A and has no obvious interactions with other residues or the substrate (Figure 3C). The lack of defined contact points in the cryo-EM structure is reflected in only weak electron density observed for the side chain of Arg20, indicating a high degree of side chain flexibility. Arg20 is positioned in a flexible loop, where it is preceded by two glycine residues, which add to its increased flexibility. Based on G20R’s position on the top rim of the β_6_ core of GCC, we suspected that it might help stabilize the interaction with the α-subunit and indirectly also facilitate CoA positioning (Figure 3C). Indeed, in mass photometry (MP) measurements, the G20R variant formed more higher mass complexes relative to GCC M5, indicating a more stable complex formation (Figure 3D,E). Thus, the higher fraction of α-subunits bound to the β_6_ core likely explains the higher *in vitro* activity of the G20R mutant.

The L100N substitution is located in the periphery of the active site, at a position that had previously been targeted during engineering efforts of GCC M5, in which this position was engineered to be serine. The Asn100 substitution is in close proximity to His143, which was proposed to coordinate the hydroxyl group of glycolyl-CoA (Figure 3F). While active site overlays of the L100N variant and GCC M5 look almost identical, we assume that Asn100 forces His143 into a more favorable rotamer conformation for substrate binding and catalysis, enabling carboxylation to occur more efficiently. This is supported by the fact that His143 is categorized as a rotamer outlier in all subunits of the L100N cryo-EM structure, which is not the case for the GCC M5 or G20R variant structures. Such minor movements of the His143 side chain toward glycolyl-CoA might facilitate improved substrate orientation/positioning and thus reduce the unfruitful decarboxylation of carboxybiotin (i.e., the release of CO_2_ from carboxybiotin without a transfer onto the substrate). This in turn decreases the reaction’s energy requirement in ATP. While the L100N variant exhibits a slightly increased proportion of higher mass oligomeric complexes in MP experiments (Figure 3G), the complex distribution remained similar to that of GCC M5 and PCC (Figure 3D,H). All investigated variants, including the PCC wild-type (Figure 3H), formed a stable β_6_ core with variable amounts of α-subunits bound in MP measurements.

## Conclusions

In this work, we demonstrated the successful application of a ML algorithm as a filtering tool to reduce the screening efforts of a random mutagenesis library of glycolyl-CoA carboxylase, GCC M5. Having trained the algorithm with 161 selected data points helped to reduce the initial sequence space of more than 10,000 sequences to only 10 candidates to screen. From these ten candidates, nine were still active in lysate-based enzyme assays and two even showed improved kinetic properties, demonstrating how screening efforts can be successfully reduced through ML-assisted strategies while increasing the fraction of positive hits at the same time.

The two newly identified GCC variants are of direct benefit for the TaCo pathway that turns photorespiration from a CO_2_-releasing into a carbon-fixing process. The reduced ATP demand of the GCC M5 L100N variant further increases the thermodynamic advantage of the TaCo pathway as a photorespiratory bypass. Note that the TaCo pathway based on the GCC M5 variant has already a minimal energy demand compared with all other natural and synthetic photosynthetic bypasses. Yet, an additional 60% reduction in ATP per carboxylation by the L100N variant would free additional ATP for biosynthetic purposes and thus further increase photosynthetic yield.^2^ We also assessed the potential side reactivity of our new GCC variants with the alternative substrate acetyl-CoA. We found that the L100N variant had a strongly decreased efficiency with that substrate (Table 1), which could be competing with glycolyl-CoA in *in vivo* settings, thus providing an additional benefit for future applications. On the other hand, the improved catalytic activity of the G20R variant provides an increased kinetic advantage. This advantage could be either direct (in case the CO_2_-fixing reaction provides a kinetic bottleneck in the TaCo pathway) or indirect (in case other enzymes of the pathway are rate limiting and require higher resource allocation). The latter advantage might generally benefit host organisms in which the burden of protein production poses a limitation. In these cases, the GCC M5 G20R variants offer the possibility to maintain a given catalytic activity *in vivo* at 2-fold lower amounts of expressed enzyme, freeing additional protein resources.

Beyond these direct effects on the TaCo pathway, we note that our strategy of augmenting screening workflows with ML-guided approaches provides an example of how enzyme and pathway engineering can profit from machine learning approaches, even for already well established (i.e., highly engineered) targets. This strategy could be generally used to develop novel biotin-dependent carboxylases (and other enzymes) for different applications, widening the scope of theoretically possible enzymes and pathways for synthetic biology applications.^18^

## Materials and Methods

### Synthesis of CoA Esters

Glycolyl-CoA was synthesized and purified as previously described.^2,3^ The concentration of CoA esters was quantified by determining the absorption at 260 nm (ε = 16.4 mM^–1^ cm^–1^) or by performing spectrophotometric substrate depletion assays.

### Random Mutagenesis Library Generation

To produce a data set to feed the ML algorithm, random mutagenesis libraries of GCC M5 were constructed. Plasmid libraries of randomly mutagenized GCC M5 were created by mega primer-based whole-plasmid PCR (MEGAWHOP).^19^ To generate randomized fragments of the β subunit of GCC M5 (pTE3101), error-prone PCR was performed using 2.5 U of Taq-polymerase with Mg-free buffer (New England Biolabs; M0320), 7 mM MgCl_2_, 0.4 mM dGTP and dATP each, 2 mM dCTP and dTTP each, 0.4 μM primer PccB_fw_P1 and primer PccB_rv_P1 each, 10% (v/v) dimethyl sulfoxide, 50 ng of template DNA of pTE3101 (see Table S2 in the Supporting Information), and 200–500 μM MnCl_2_ in a 50 μL reaction. The randomized fragments were digested with *Dpn*I (NEB, R0176), purified by agarose gel electrophoresis, and used as mega primers for a whole-plasmid PCR, as described elsewhere,^19^ or subjected to another error-prone PCR reaction to further increase the mutation rate. The MEGAWHOP reaction (50 μL) contained 1× KOD Hot Start reaction buffer (Novagen), 0.2 mM dNTPs, 1.5 mM MgSO_4_, 500 ng of mega primer, 50 ng of template plasmid (GCC M5; pTE3101), and 2.5 U of KOD Hot Start DNA polymerase (Novagen). The MEGAWHOP product was purified, digested with *Dpn*I, and transformed into ElectroMAX DH5α (Invitrogen) to ensure a high number of transformants in the resulting libraries. To estimate the mutation rate for the different concentrations of MnCl_2_ used in the error-prone PCR, the plasmids of ten randomly picked clones after MEGAWHOP were purified, sequenced, and analyzed for nucleotide exchanges.

### Protein Production and Purification

For the production and purification of GCC M5 and its variants, the corresponding plasmid was transformed into chemically competent *E. coli* BL21-*birA* cells (see Table S1 in the Supporting Information). Cells were grown on lysogeny broth (Miller recipe) agar plates containing 100 μg/mL ampicillin and 50 μg/mL spectinomycin at 25 °C overnight. Eight liters of lysogeny broth (Miller recipe) containing 5 g/L yeast extract, 10 g/L tryptone, 10 g/L NaCl, 17 mM KH_2_PO_4_, 72 mM K_2_HPO_4_, and 0.4% glycerol were inoculated from the agar plate and incubated at 37 °C and 140 rpm. At OD_600_ = 0.4–0.6, protein expression was induced with 500 μM IPTG and cells were incubated overnight at 25 °C. Cell harvesting at 8000*g* and 4 °C for 12 min and lysis by French Pressing at 137 MPa was followed by centrifugation at 100,000 *g* and His-Trap purification using an Äkta Start (GE Healthcare) with a HisTrap FF column (GE Healthcare). The purification buffer contained 50 mM HEPES, pH 7.8, and 500 mM KCl, and the elution was done with 500 mM imidazole. Protein desalting occurred via gel filtration chromatography using a HiLoad 16/600 Superdex 200 pg column (GE Healthcare) and a buffer containing 50 mM HEPES, pH 7.8, and 150 mM KCl. Protein quantification occurred by an absorbance measurement at 280 nm. Protein purity was validated by SDS-PAGE using 15 μg of purified protein on a 4–20% Mini-Protean TGX Precast Protein Gel (Biorad).

### Enzyme Assays

Enzyme activity assays were performed in three different ways. Screening of randomly mutagenized GCC to produce a data set to train an ML algorithm occurred via lysate-based measurements in plate readers. Prescreening of variants that were predicted by the ML algorithm and selected by homology modeling and structural analysis was done with the same assay. The determination of carboxylation rates and ATP per carboxylation (ATP/CO_2_) ratios occurred via spectrophotometric measurements with purified enzymes.

### Lysate-Based Measurements of Carboxylation Rate and ATP-Hydrolysis

GCC-encoding constructs, random-mutagenesis libraries of GCC, or site-saturation mutagenesis libraries of GCC were transformed into *E. coli* BL21_*birA* (see Table S1 in the Supporting Information), and colonies were picked into 96-deep-well plates (PlateOne) with lysogeny broth (Miller recipe) containing 100 μg/mL ampicillin and 50 μg/mL streptomycin. The plates were incubated overnight at 37 °C with subsequent transfer into fresh 96-deep-well plates with lysogeny broth (Miller), 100 μg/mL ampicillin, 50 μg/mL spectinomycin, and 2 μg/mL biotin to an OD_600_ of 0.1. Protein expression was induced with 0.25 mM isopropyl β-d-1-thiogalactopyranoside (IPTG) at an OD_600_ of 0.4–0.6, and the cells were incubated overnight at 25 °C. The cells were lysed using CelLytic B (Sigma–Aldrich) and stored in 20% glycerol at −80 °C. The enzyme activity was measured in a plate reader by the coupled enzyme assay with purified malonyl-CoA reductase from *Chloroflexus aurantiacus* (later referred to as CaMCR; E.C. 1.1.1.298 and E.C. 1.2.1.75) as described earlier.^2^ We used small-volume 384-well plates (Greiner Bio-One) with 2 μL of cell extract, 100 mM 3-(*N*-morpholino)propanesulfonic acid (MOPS), pH 7.8, 1 mM ATP, 50 mM KHCO_3_, 500 μg/mL CaMCR, 1 mM NADPH, 10 mM MgCl_2_, and 1 mM glycolyl-CoA in a reaction volume of 10 μL. The absorbance of NADPH was measured at 340 nm and 37 °C for 5 h with intervals of 47 s in a plate reader (Tecan Infinite M Plex).

### Spectrophotometric Measurements of Carboxylation Rate

To measure the carboxylation rate of GCC, a coupled spectrophotometric enzyme assay with CaMCR was performed. 100 mM MOPS, pH 7.8, 50 mM KHCO_3_, 2 mM ATP, 0.3 mM NADPH, 5 mM MgCl_2_, 1.8 mg/mL CaMCR from *Chloroflexus aurantiacus*, and 0.01–1 mg/mL GCC were mixed in a cuvette and incubated for 2 min at 37 °C. The reaction was started with 0.5 mM glycolyl-CoA, and absorption was measured over time at λ = 340 nm.
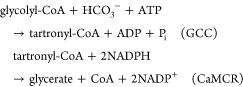


### Spectrophotometric Measurements of ATP Hydrolysis

To measure the ratio between ATP consumption and carboxylation of GCC, a coupled enzyme assay with CaMCR under ATP-limited conditions was performed. 100 mM MOPS, pH 7.8, 50 mM KHCO_3_, 0.15 mM ATP, 0.5 mM NADPH, 5 mM MgCl_2_, 1.8 mg/mL CaMCR from *Chloroflexus aurantiacus*, and 0.05–3 mg/mL GCC were mixed in a cuvette and incubated for 2 min at 37 °C. The reaction was started with 0.5 mM glycolyl-CoA, and absorption was measured over time at λ = 340 nm. The ATP per carboxylation ratio for glycolyl-CoA carboxylation was calculated from the ratio between the ATP amount in the reaction mixture and the consumed amount of NADPH that is reflected by the absorbance drop during the reaction.
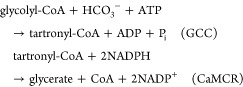


### Mass Spectrometry of CoA Esters

Quantitative determination of CoA esters was performed using a LC-MS/MS. The chromatographic separation was performed on an Agilent Infinity II 1290 HPLC system using a Kinetex EVO C18 column (150 mm × 2.1 mm, 3 μm particle size, 100 Å pore size, Phenomenex) connected to a guard column of similar specificity (20 mm × 2.1 mm, 3 μm particle size, Phenomenex) a constant flow rate of 0.25 mL/min with mobile phase A being 50 mM ammonium acetate in water at a pH of 8.1 and phase B being 100% methanol (Honeywell, Morristown, New Jersey, USA) at 25 °C.

The injection volume was 1 μL. The mobile phase profile consisted of the following steps and linear gradients: 0–2 min constant at 0% B; 2–5 min from 0 to 6% B; 5–8 min from 6 to 23% B; 8–10 min from 23 to 80% B; 10–11 min constant at 80% B; 11–12 min from 80 to 0% B; 12 to 18 min constant at 0% B. An Agilent 6495 ion funnel mass spectrometer was used in positive mode with an electrospray ionization source and the following conditions: ESI spray voltage 1000 V, nozzle voltage 1000 V, sheath gas 400 °C at 11 L/min, nebulizer pressure 20 psig, and drying gas 100 °C at 11 L/min. Compounds were identified based on their mass transition and retention time compared to standards. Chromatograms were integrated by using MassHunter software (Agilent, Santa Clara, CA, USA). Relative abundance was determined based on the peak area, and absolute concentrations were determined based on an external standard curve.

Mass transitions, collision energies, cell accelerator voltages, and dwell times have been optimized using chemically pure standards. Parameter settings of all targets are given in Table 2.

**Table 2:** Parameters for LC-MS/MS

name	precursor ion	product ion	collision energy [V]	fragmentor voltage [V]	cell accelerator voltage [V]	polarity
tartronyl-CoA	870.2	428.1	31	380	5	positive
tartronyl-CoA	870.2	319	40	380	5	positive
methylmalonyl-CoA	868.1	428.1	37	380	5	positive
methylmalonyl-CoA	868.1	317.1	41	380	5	positive
glycolyl-CoA	826.1	428.1	28	380	5	positive
glycolyl-CoA	826.1	319.1	33	380	5	positive
propionyl-CoA	824.1	428.1	28	380	5	positive
propionyl-CoA	824.1	317.1	31	380	5	positive

### Machine Learning Model Creation and Training

We developed a ML algorithm using reproducing kernel Hilbert space methods. In particular, we encoded the *j*th sequence as a vector **v**_*j*_ residing in a reproducing kernel Hilbert space. These vectors are the basis for a Gaussian Process (GP) regression model to predict enzymatic properties such as catalytic speed and ATP efficiency.^17^ To define the kernels used for encoding an amino acid sequence into a vector, we considered both classical sequence kernels such as kernels based on hamming distance and alignment kernels.^20^ The final model uses a kernel that compares positions of two sequences based on a BLOSUM62 matrix and a regularization constant of 1.0 for the inversion of the gram matrix for the GP.

As a comparison model and a baseline, we adopted the approach of Ma et al.^15^ This approach uses the Uniref 1900 model (Unified Rational Protein Engineering with Sequence-Based Deep Representation Learning) that embeds protein sequences into a 1900-dimensional real vector space simply by averaging the hidden unit activations of a Long Short-Term Memory model. This model was pretrained on Uniref50 sequences in a language-modeling task, i.e., given the start of the sequence, the next amino acid in the sequence had to be predicted. The 1900-dimensional embedding as present in the jax-unirep package was used as the basis for a random forest regressor fitted with scikit-learn using default settings. This is similar to the approach of Hsu et al., which also combines model-based learning of the structure of evolutionary conservation with a supervised learning approach.^21^

Both used models were multitask models and predicted three targets: carboxylation rate, ATP demand, and a combination of both for the purpose of computing a unified decision criterion for the suggestions. The Supporting Information gives a more detailed description of the combined performance measure. The two models were evaluated in a 10-fold cross validation scheme on the 161 unique enzyme sequences with measured carboxylation rates and ATP per carboxylation ratios (90%–10% split, corresponding to 140 sequences in train, 16 in validation). The rank correlation between the upper confidence bound of the GP and ground truth was ρ = 0.42, and between the Unirep+random forest prediction and ground truth, it was ρ = 0.39 (Kendalls rank correlation was τ = 0.28 for GP, τ = 0.26 for RF). Typical quality metrics for regression models such as mean squared error are not of interest in our setting since we are looking to prioritize candidates for subsequent experiments rather than build a regression model with high accuracy. Rank correlation is the metric that captures this best. It was not necessary to employ hyperparameter search for the two models, since the average rank correlation of ρ = 0.42 for the GP model already promised strong improvements compared to the error-prone PCR protocol used so far.

The basic premise of the upper confidence bound criterion that we used for candidate prioritization is that a sequence (or input coordinate) should be synthesized and measured (chosen for evaluation) if it has a good chance of maximizing measured activity and ATP efficiency (the property of interest). The upper confidence bound of the *j*th candidate sequence is computed as μ_*j*_ + βσ_*j*_ where μ_*j*_ and σ_*j*_ are the predictive mean and standard deviation of the GP model, and β is a parameter between 0 and 1 encoding the exploitation–exploration trade off and was set to 0.5.

In order to obtain a combined performance metric, the training data for the carboxylation rate and ATP demand were normalized to have zero mean and unit variance, ensuring comparability between them. They were then added to create a single performance metric encapsulating both properties. Although this approach may not be ideal for multiobjective Bayesian Optimization, it mimics standard techniques in machine learning, where multiple loss functions and/or regularizers are summed to address various objectives. The Upper Confidence Bound (UCB) of this combined performance metric was employed to rank the candidate list.

### Structural Modeling and Analysis

To assess mutations that were predicted by the ML algorithm, homology modeling of variants in the top 1% predictions was performed using SWISS-MODEL. As a template for homology modeling of GCC mutations, the structure of the engineered GCC M5 from *Methylorubrum extorquens* (PDB 6YBQ) was used. Structural analysis of the models was done using PyMOL (the PyMOL Molecular Graphics System; version 2.5.7; Schrödinger). Modeling of glycolyl-CoA into the active site of GCC was based on the positions of CoA in the GCC M5 structure and methylmalonyl-CoA in the structure of a methylmalonyl-CoA carboxytransferase from *Propionibacterium freudenreichii* (PDB 1ON3; 52% amino acid identity). Manual fitting of the glycolyl-CoA reflecting differences in active-site architectures was done in the *Coot* (0.9.8.3) and PyMOL programs.

### Site-Directed Mutagenesis

Site-directed mutagenesis was used to construct variants of GCC that were earlier predicted by the ML algorithm and selected by homology modeling and structural analysis. The introduction of novel mutations was done by single mutagenic oligonucleotide PCR as described elsewhere.^22^ A 25 μL reaction mixture containing 0.5 μM primer, 3% (v/v) dimethyl sulfoxide, 50 ng of template DNA (pTE3101), and Phusion High-Fidelity PCR Master Mix (NEB, M0531) was used for PCR and subsequently digested with *Dpn*I (NEB, R0176) by adding 20 U to the reaction mixture and incubating 2 h at 37 °C. Five microliters were transformed into chemically competent *E. coli* NEB Turbo cells, which were streaked out on lysogeny broth (Miller) agar plates with 50 μg/mL streptomycin. Three to six colonies were picked and cultivated in 10 mL of lysogeny broth (Miller) with 50 μg/mL streptomycin for 12 h at 37 °C and 180 rpm, and finally, the plasmids were isolated and sequenced to validate the mutagenesis.

### Site-Saturation Mutagenesis

Plasmid libraries of GCC with defined residues to be saturated with all amino acids were created by whole plasmid PCR with primer mixes containing different base edits. Primers were designed with the 22c-trick to have reduced codon redundancy.^23^ For the whole plasmid PCR, forward primers were mixed in a 12:9:1 ratio to achieve equal amounts of each primer (Table S3). A 50 μL reaction mixture containing 0.5 μM primer, 3% (v/v) dimethyl sulfoxide, 100 ng of template DNA (pTE3101), and Phusion High-Fidelity PCR Master Mix (NEB, M0531) was used for PCR and subsequently digested with *Dpn*I (NEB, R0176) by adding 20 U to the reaction mixture and incubating 2 h at 37 °C. After a PCR-clean up, 5 μL was transformed into chemically competent *E. coli* NEB Turbo cells and streaked out on lysogeny broth (Miller) agar plates with 50 μg/mL streptomycin. Colonies were flushed from the plate and plasmids were isolated. To ensure coverage of all plasmid variants in the libraries, at least 1300 colonies were collected, representing a 65-fold oversampling. Codon diversity was confirmed by sequencing of the library.

### Mass Photometry

Mass photometry measurements were carried out on microscope coverslips (1.5 H, 24 mm × 50 mm, Carl Roth) with CultureWell Reusable Gaskets (CW-50R-1.0, 50 3 mm diameter × 1 mm depth) that had been washed by three consecutive rinses of water and isopropanol, prior to drying under a stream of pressurized air. Gaskets were assembled on microscope coverslips and placed on the stage of a TwoMP mass photometer (MP, Refeyn Ltd., Oxford, UK) with immersion oil. Measurements were carried out in 1× phosphate-buffered saline (PBS, 10 mM Na_2_HPO_4_, 1.8 mM KH_2_PO_4_, 137 mM NaCl, 2.7 mM KCl (pH 7.4)). To this end, 18 μL of 1× PBS was used to focus the MP before 2 μL of sample (1 μM protein) was added, rapidly mixed, and measured. Shortly before measuring, samples were prepared by diluting purified protein to 1 μM monomer concentration in buffer (50 mM HEPES, pH 7.8, and 150 mM KCl), as determined by absorption at 280 nm. Data acquisition was done for 60 s at 100 frames per second using AcquireMP (Refeyn Ltd., Oxford, UK). Mass photometry contrast was calibrated to molecular masses using 50 nM in-house purified protein mixture containing citrate-synthase complexes of known molecular masses ranging from 86 to 430 kDa. Mass photometry data sets were processed and analyzed using DiscoverMP (Refeyn Ltd., Oxford, UK). Details of mass photometry image analysis have been described previously.^24^

### CryoEM Sample Preparation and Data Collection

Three microliters of protein solution (1 mg/mL) in 50 mM HEPES, pH 7.8, and 150 mM KCl containing 2 mM MgCl_2_, 1 mM ATP, and 4 mM glycolyl-CoA were applied to QUANTIFOIL R2/1 300 copper mesh grids that were glow-discharged for 90 s immediately before use and blotted for 3.5 s with blot force 4 at 100% humidity and 4 °C using a Vitrobot Mark IV (Thermo Scientific). Grids were plunge frozen in liquid ethane cooled by liquid nitrogen and used for data collection immediately.

CryoEM data were acquired on a Titan Krios G3i electron microscope (Thermo Scientific), operated at an acceleration voltage of 300 kV and equipped with a BioQuantum-K3 imaging filter (Gatan). Data were measured in electron counting mode at a nominal magnification of 105,000× (0.837 Å/pixel) with a total dose of 55 e^–^/A^2^ (55 fractions), using the aberration-free image-shift (AFIS) correction in EPU (Thermo Scientific). Five images were acquired per foil hole, and the nominal defocus range for data collection was −0.5 to −2.0 μm.

### CryoEM Data Processing

Data sets were processed entirely in CryoSPARC (version 4.1 or 4.2).^25^ For all data sets dose-fractionated movies were gain-normalized, aligned, and dose-weighted using Patch Motion correction. The contrast transfer function (CTF) was determined by using the Patch CTF routine. Information regarding cryoEM data collection, model refinement, and statistics are listed in Table S5.

### Processing of GCC M5 G20R

Blob picker and manual inspection of particles were used to extract an initial 837,101 particles with a box size of 256 pixels, which were used to build 2D classes. 2D classes with protein-like features were used to initialize template picking. After inspection and extraction with a box size of 256 pixels, this yielded 3,439,715 particles, which were used to build 2D classes. A total of 1,845,969 candidate particles were selected from 2D classes and used for *ab initio* reconstruction and classification into 4 classes. Particles of the best-aligning class (647,870 particles) were subjected to nonuniform with per-particle defocus optimization, per-group CTF parameter optimization, and EWS correction. This yielded a map with a 2.08 Å global resolution and a temperature factor of 66.1 Å^2^, which was subsequently locally refined to yield a map with 2.03 Å global resolution and a temperature factor of 58.9 Å^2^. The resulting map was B-factor sharpened by −40 Å^2^. Further classification did not yield improved resolution.

### Processing of GCC M5 L100N

Blob picker and manual inspection of particles were used to extract an initial 620,147 particles with a box size of 500 pixels, which were used to build 50 2D classes. 2D classes with protein-like features were used to initialize template picking. After inspection and extraction with a box size of 500 pixels, this yielded 2,511,911 particles, which were used to build 2D classes. A total of 324,129 candidate particles were selected and used for *ab initio* reconstruction and classification into 5 classes. Particles of the best-aligning class (113,824 particles) were subjected to nonuniform refinement with per-particle defocus optimization, per-group CTF parameter optimization, and EWS correction. This yielded a map with a 2.36 Å global resolution and a temperature factor of 66.9 Å^2^, which was subsequently locally refined to yield a map with 2.31 Å global resolution and a temperature factor of 60.6 Å^2^. The resulting map was B-factor sharpened by −50 Å^2^. Further classification did not yield improved resolution.

### Model Building and Refinement

CryoEM map fitting was initially performed in UCSF-ChimeraX (v1.6)^26^ using GCC M5 (PDB 6YBQ) as template. The resulting model was manually built further in *Coot* (v0.9.8.3).^27^ Automatic refinement of the structure was performed using phenix.real_space_refine of the Phenix (v1.20.1) software suite.^28^ Manual refinements and water picking were performed in *Coot*. The model statistics are listed in Table S5.

## Supporting Information Available

Supplementary methods; strains, plasmids, and oligonucleotides used in this study; selected GCC candidates based on ML-prediction; cryoEM data collection, refinement, and model statistics; spectrophotometric measurements of selected GCC variants; lysate-based screen of random mutagenesis library and of preselected variants; ML-predicted enzyme properties and experimentally measured properties; cryo-EM data collection and analysis for GCC M5 G20R and GCC M5 L100N; Michaelis–Menten kinetics for GCC M5 G20R and L100N (PDF)
Supplementary file 1, screening data and sequence list for model feeding (XLSX)
Supplementary file 2, machine learning prediction table (XLSX)
