# Supplementary material for: Machine Learning-Supported Enzyme Engineering toward Improved CO2-Fixation of Glycolyl-CoA Carboxylase
Source: ACS Synth Biol. 2023 Nov 20;12(12):3521–30. doi: 10.1021/acssynbio.3c00403 (PMC10729300; doi:10.1021/acssynbio.3c00403)
Supplement: Supplementary file 1 — sb3c00403_si_001.pdf [file sb3c00403_si_001.pdf]

Supporting Information

Machine learning-supported enzyme engineering towards improved CO_2-fixation of glycolyl-CoA carboxylase

Daniel G. Marchal[a], Luca Schulz[a], Ingmar Schuster[b], Jelena Ivanovska[b], Nicole Paczia[c], Simone Prinz[d], Jan Zarzycki[a], Tobias J. Erb[ae*]

* Correspondence should be addressed to: T.J.E. (toerb@mpi-marburg.mpg.de)

[a]Department of Biochemistry and Synthetic Metabolism, Max-Planck-Institute for Terrestrial Microbiology, Marburg, Germany; [b]Exazyme GmbH, Berlin, Germany; [c]Core Facility for Metabolomics and Small Molecule Mass Spectrometry, Max-Planck-Institute for Terrestrial Microbiology, Marburg, Germany; [d]Central Electron Microscopy Facility, Max-Planck-Institute of Biophysics, Frankfurt, Germany; [e]SYNMIKRO Center for Synthetic Microbiology, Marburg, Germany

Contents

Supplementary methods

Table S1. Strains used in this study.

Table S2. Plasmids used in this study.

Table S3. Oligonucleotides used in this study.

Table S4. Selected GCC candidates based on ML-prediction table.

Table S5. CryoEM data collection, refinement, and model statistics.

Table S6. Spectrophotometric measurements of selected GCC variants.

Figure S1. Lysate-based screen of random mutagenesis library.

Figure S2. ML-predicted enzyme properties and experimentally measured properties.

Figure S3. Lysate-based screen of preselected mutant variants.

Figure S4. Cryogenic electron microscopy (Cryo-EM) data collection and analysis for GCC M5 G20R.

Figure S5. Cryogenic electron microscopy (Cryo-EM) data collection and analysis for GCC M5 L100N.

Figure S6. Michaelis-Menten kinetics for GCC M5 G20R and L100N.

Supplementary methods

Materials

Chemicals were obtained from Sigma-Aldrich, Carl Roth GmbH + Co. KG, Santa Cruz Biotechnology Inc. and Merck. Biochemicals and materials for cloning and protein expression were obtained from Thermo Fisher Scientific, New England Biolabs GmbH and Macherey-Nagel GmbH. Coenzyme A was bought from Roche Diagnostics. Materials and equipment for protein purification were obtained from GE Healthcare, BioRad and Merck Millipore GmbH. Pyruvate Kinase/Lactic Dehydrogenase, Malic Dehydrogenase, Glucose-6-Phosphate Dehydrogenase, Glucose Dehydrogenase and Phosphoenolpyruvate carboxylase were bought from Sigma-Aldrich.

Strains

All strains used in this work are listed in the following table.

Table S1. Strains used in this study.

Strain	Genotype/Phenotype	Reference
E. coli NEB Turbo	F' *proA*$^{+}$*B*$^{+}$ *lacI*^{q} Δ*lacZM15* / *fhuA2* Δ*(lac-proAB)* *glnV* *galK16* *galE15* *R(zgb-210::Tn10)*Tet^{S} *endA1* *thi-1* Δ*(hsdS-mcrB)5*	NEB
E. coli BL21 DE3	*fhuA2 [lon] ompT gal (λ DE3) [dcm]* Δ*hsdS* *λ DE3 = λ sBamHIo* Δ*EcoRI-B int::(lacI::PlacUV5::T7 gene1) i21* Δ*nin5*	NEB
E. coli BL21-*birA*	*fhuA2 [lon] ompT gal (λ DE3) [dcm]* Δ*hsdS* *λ DE3 = λ sBamHIo* Δ*EcoRI-B int::(lacI::PlacUV5::T7 gene1) i21* Δ*nin5 amp*^{R} *harbors JZ150 with birA from M. extorquens*	[1]
E. coli ElectroMAX DH5α	F^{-} Φ80*lac*ZΔM15 Δ(*lac*ZYA-*arg*F) U169 *rec*A1 *end*A1 *hsd*R17 (r_k^-, m_k^+) *gal*$^{-}$ *pho*A *sup*E44 λ^{-} *thi*$^{-}$1 *gyr*A96 *rel*A1	Invitrogen

E. coli ElectroMAX DH5α was used to create random mutagenesis libraries that were needed to produce a dataset of randomly mutagenized GCC variants to train a machine learning model for the prediction of beneficial mutations. *E. coli* NEB Turbo was used to construct and maintain plasmids with site-specific mutations in the gene for GCC. *E. coli* BL21-*birA* was derived from *E. coli* BL21 DE3 by introducing a vector that bears a biotin ligase gene from Methylorubrum extorquens that is required to activate GCC. *E. coli* BL21-birA was used for protein overexpression of GCC variants.

Plasmids

All plasmids used in this work are listed in the following Table S2.

Table S2. Plasmids used in this study.

**Plasmid**	**Description**	**Reference**
pTE3101	Glycolyl-CoA carboxylase M5, adapted from *M. extorquens* PCC, codon optimized for *E. coli*	[1]
pTE3142	Glycolyl-CoA carboxylase M5_L8F, adapted from *M. extorquens* PCC, codon optimized for *E. coli*	This work
pTE3143	Glycolyl-CoA carboxylase M5_G20R, adapted from *M. extorquens* PCC, codon optimized for *E. coli*	This work
pTE3144	Glycolyl-CoA carboxylase M5_M53E, adapted from *M. extorquens* PCC, codon optimized for *E. coli*	This work
pTE3145	Glycolyl-CoA carboxylase M5_M53Q, adapted from *M. extorquens* PCC, codon optimized for *E. coli*	This work
pTE3146	Glycolyl-CoA carboxylase M5_M64R, adapted from *M. extorquens* PCC, codon optimized for *E. coli*	This work
pTE3147	Glycolyl-CoA carboxylase M5_L100N, adapted from *M. extorquens* PCC, codon optimized for *E. coli*	This work
pTE3148	Glycolyl-CoA carboxylase M5_D407V, adapted from *M. extorquens* PCC, codon optimized for *E. coli*	This work
pTE3149	Glycolyl-CoA carboxylase M5_T495N, adapted from *M. extorquens* PCC, codon optimized for *E. coli*	This work
pTE3150	Glycolyl-CoA carboxylase M5_W502H, adapted from *M. extorquens* PCC, codon optimized for *E. coli*	This work
pTE3151	Glycolyl-CoA carboxylase M5_L510F, adapted from *M. extorquens* PCC, codon optimized for *E. coli*	This work
pTrc-McrCa	Malonyl-CoA reductase from *Chloroflexus aurantiacus*, codon optimized for *E. coli*	[2]

Oligonucleotides

All oligonucleotides used in this work are listed in the following Table S3.

Table S3. **Oligonucleotides used in this study.**

Oligo	Description	Sequence (5' → 3')
oDM0088	Sequencing of GCC constructs	cgtccacgaaggtgacgatcg
oDM0115	GCC M5 substitution L8F (ctt to ttt)	ccatatgaaggacatcctcgagaagtttgaggagcgtcgcgcacagg
oDM0116	GCC M5 substitution G20R (ggg to cgt)	gcacaggcccgtctcggcggccgtgaaaagcggctcgagg
oDM0117	GCC M5 substitution M53E (atg to gaa)	cacgggtcgttcgaggagttcgacgaattcgtgcagcaccgctccaccg
oDM0118	GCC M5 substitution M53Q (atg to cag)	cacgggtcgttcgaggagttcgaccagttcgtgcagcaccgctccaccg
oDM0119	GCC M5 substitution M64R (atg to cgt)	gcagcaccgctccaccgatttcggccgtgagaagcagaagatccccggcg
oDM0120	GCC M5 substitution S100N (tcc to aac)	cacggtgttcggcggctcgaactccgaggcgcacgcagccaag
oDM0121	GCC M5 substitution I407V (atc to gtc)	caaggccttcggcggcgcctacgtcgtcatggcctccaagcatgtcggcg
oDM0122	GCC M5 substitution T495N (acc to aac)	gggcgctcgggatgctgcgcaacaaggagatggagcagcccagg
oDM0123	GCC M5 substitution R502H (agg to cat)	gcaccaaggagatggagcagccccataagaagcacgacaacatcccgctc
oDM0124	GCC M5 substitution L510F (ctc to ttc)	cccaggaagaagcacgacaacatcccgttctgagaattcgagctccgtcg
oDM0164	GCC M5 G20X site saturation primer 1	gcacaggcccgtctcggcggcndtgaaaagcggctcgagg
oDM0165	GCC M5 G20X site saturation primer 2	gcacaggcccgtctcggcggcvhggaaaagcggctcgagg
oDM0166	GCC M5 G20X site saturation primer 3	gcacaggcccgtctcggcggctgggaaaagcggctcgagg
oDM0167	GCC M5 L100X site saturation primer 1	cacggtgttcggcggctcgndttccgaggcgcacgcagccaag
oDM0168	GCC M5 L100X site saturation primer 2	cacggtgttcggcggctcgvhgtccgaggcgcacgcagccaag
oDM0169	GCC M5 L100X site saturation primer 3	cacggtgttcggcggctcgttgtccgaggcgcacgcagccaag
oDM0170	GCC M5 G20X site saturation primer 4	gcgacgctcctcaagcttc
oDM0171	GCC M5 L100X site saturation primer 4	aagtccttcgagaacaggaagac
PCC_seq1	Sequencing of GCC constructs	gagcggataacaattcccctg
PCC_seq2_II	Sequencing of GCC constructs	ccaggacatggcgctgaag
PCC_seq3_II	Sequencing of GCC constructs	gaaggcgacttcttcgagatc
PCC_seq4	Sequencing of GCC constructs	gatgctgcgcaccaag
PCC_seq5	Sequencing of GCC constructs	caatccgggtgccatc
PCC_seq6	Sequencing of GCC constructs	gaacacccgcctgcag
PCC_seq7	Sequencing of GCC constructs	cgatcgatcacaagctcaac
PccB_fw_P1	Random mutagenesis primer	gtttaactttaataaggagatataccatgggcagcagccatc
PccB_rv_P1	Random mutagenesis primer	gattactttctgttcgacttaagcattatgcggccgcaag

Table S4. Selected GCC candidates based on ML-prediction.

Candidate[a]	Rank	Top ratio	Mutations versus PCC[2]	Justification
**G20R**	3/10019	0.03 %	**G20R**, L100S, Y143H, D407I, I450V, W502R	Mutations at this position are listed three times in the top 1.0 % of predicted mutations. Arg20 sits on the surface in the proximity to the CoA phosphate group, it could pull it away or push it into the active site.
**L8F**	5/10019	0.05 %	**L8F**, L100S, Y143H, D407I, I450V, W502R	Leu8 sits in a hydrophobic pocket. Phe at that position could form hydrophobic interactions with neighboring residues and hence affect the protein folding.
**M53Q**	20/10019	0.20 %	**M53Q**, L100S, Y143H, D407I, I450V, W502R	Mutations at this position are listed nine times in the top 1.0 % of predicted mutations. Gln53 could form a hydrogen bond to the neighboring subunit.

				Additionally Gln53 is more common than Met53 in homologous proteins.
**M53E**	26/10019	0.26 %	**M53E**, L100S, Y143H, D407I, I450V, W502R	Mutations at this position are listed nine times in the top 1.0 % of predicted mutations. Glu53 could form a hydrogen bond to the neighboring subunit. Additionally Glu53 is more common than Met53 in homologous proteins.
**L510F**	46/10019	0.46 %	L100S, Y143H, D407I, I450V, W502R, **L510F**	Mutations at this position are listed three times in the top 1.0 % of predicted mutations. This residue sits in a very hydrophilic pocket and Phe510 has more possible interactions than Leu510.
**T495N**	102/10019	1.02 %	L100S, Y143H, D407I, I450V, **T495N**, W502R	This residue is surface exposed and sits at the interface to two other subunits. Asn495 has more possible interactions and probably a higher solubility than Thr495.
**M64R**	104/10019	1.04 %	**M64R**, L100S, Y143H, D407I, I450V, W502R	This residue sits in proximity to the CoA-phosphate group. Arg64 could have a positive impact on the CoA positioning by interacting with the phosphate group.
**L100N**	303/10019	3.02 %	**L100N**, Y143H, D407I, I450V, W502R	This mutation was already tested in the background of GCC M2. Scheffen et al. (2021) showed that this residue sits in the active site and has an impact on catalysis.[1] GCC M5 bears L100S mutation.
**W502H**	382/10019	3.81 %	L100S, Y143H, D407I, I450V, **W502H**	This residue was already mutated earlier but not to His. Scheffen et al. (2021) showed that this residue has impact on substrate binding.[1] GCC M5 bears W502R mutation.
**D407V**	410/10019	4.09 %	L100S, Y143H, **D407V**, I450V, W502R	This residue was already mutated earlier but not to Val. Scheffen et al. (2021) showed that this residue has an impact on substrate binding.[1] GCC M5 bears D407I mutation. V407 is common in homologous proteins.

[a]Candidates are labeled after the substitution that distinguishes them from GCC M5. Positions of substitutions correspond to PCC from *M. extorquens.* [2]All GCC variant substitutions are listed versus the PCC from *M. extorquens*.

Cryo-EM

Table S5. CryoEM data collection, refinement, and model statistics.

**Data collection**		
Model	GCC M5 G20R	GCC M5 L100N
Accessions	PDB 8PN7, EMD-17777	PDB 8PN8, EMD-17778
Microscope	Titan Krios G3i	Titan Krios G3i
Voltage (kV)	300	300
Camera	K3	K3
Magnification	105,000	105,000
Pixel size at detector (Å/pixel)	0.837	0.837
Total electron exposure ($e^-/Å^2$)	55	55
Exposure rate (e^-/pixel/sec)	15	15
Frames per exposure	55	55
Defocus range (µm)	-0.5 to -2.0	-0.5 to -2.0
Automation software	EPU, CryoSPARC	EPU, CryoSPARC
Energy filter slit width	-30 eV	-30 eV
Micrographs collected (no.)	9713	9783
Micrographs used (no.)	9713	9783
Total extracted particles (no.)	3,439,715	2,511,911
Refined particles (no.)	647,870	113,824
Final particles (no.)	647,870	113,824
Point-group	D3	D3
Resolution (global, Å)	2.03	2.31
$FSC_{0.143}$	2.60 / 2.03 (unmasked / masked)	3.2 / 2.31 (unmasked / masked)
Resolution range (local, Å)	1.85 – 10.31	1.83 – 28.83
Map sharpening B-factor ($Å^2$)	-40.0	-50.0
Map sharpening methods	CryoSPARC sharpening	CryoSPARC sharpening
**Model Refinement**		
Refinement package	Phenix	Phenix
method	real space	real space
resolution cutoff	2.05	2.36
Protein residues	4308	4314
Ligands	6× CoA, 6× biotin	6× CoA, 6× biotin
Model-Map scores		
CC_{Map}	0.76	0.78
avg. $FSC_{0.143}$	2.05 / 1.97 (unmasked / masked)	2.35 / 2.08 (unmasked / masked)
B-factors ($Å^2$) (min / max / mean)		
Protein residues	1.95 / 140.39 / 54.63	4.25 / 173.70 / 62.54
Ligands<br>Waters	71.73 / 101.63 / 84.38<br>22.85 / 80.84 / 52.66	78.91 / 115.73 / 95.36<br>29.22 / 93.26 / 51.68
R.M.S. Bond lengths (Å)	0.002	0.002
R.M.S. Bond angles (°)	0.464	0.432
**Validation**		
MolProbity score	1.22	1.22
CaBLAM outliers	1.90	1.71
Clashscore	4.47	4.39
Poor rotamers (%)	0.35	0.43
C-beta deviations	0.00	0.00
EMRinger score	5.80	5.17
Ramachandran favored (%)	98.53	98.97
Ramachandran outliers (%)	0.00	0.00

Enzyme kinetics

Table S6. Spectrophotometric measurements of selected GCC variants.

Candidate[a]	Specific activity [nmol min^{-1} mg^{-1}][b]	ATP / carboxylation ratio
**GCC M5 (reference)**	940 ± 40	4.00 ± 0.02
**L8F**	680 ± 50	4.40 ± 0.06
**G20R**	**2600 ± 430**	4.36 ± 0.11
**M53E**	n.d.	n.m.
**M53Q**	n.d.	n.m.
**M64R**	n.d.	n.m.
**L100N**	640 ± 70	**1.71 ± 0.11**
**D407V**	640 ± 80	6.65 ± 0.08
**T495N**	410 ± 70	4.45 ± 0.34
**W502H**	630 ± 55	4.96 ± 0.21
**L510F**	n.d.	n.m.

[a]Candidates are labeled after the mutation that distinguishes them from GCC M5. The positions of the substitutions are corresponding to the original PCC of *M. extorquens*. [b]Measured at 0.5 mM glycolyl-CoA and 37 °C. n.d. = not detectable, n.m. = not measured, n = 6 for GCC M5, G20R and L100N, n = 3 for all other variants.

Supplemental Figures

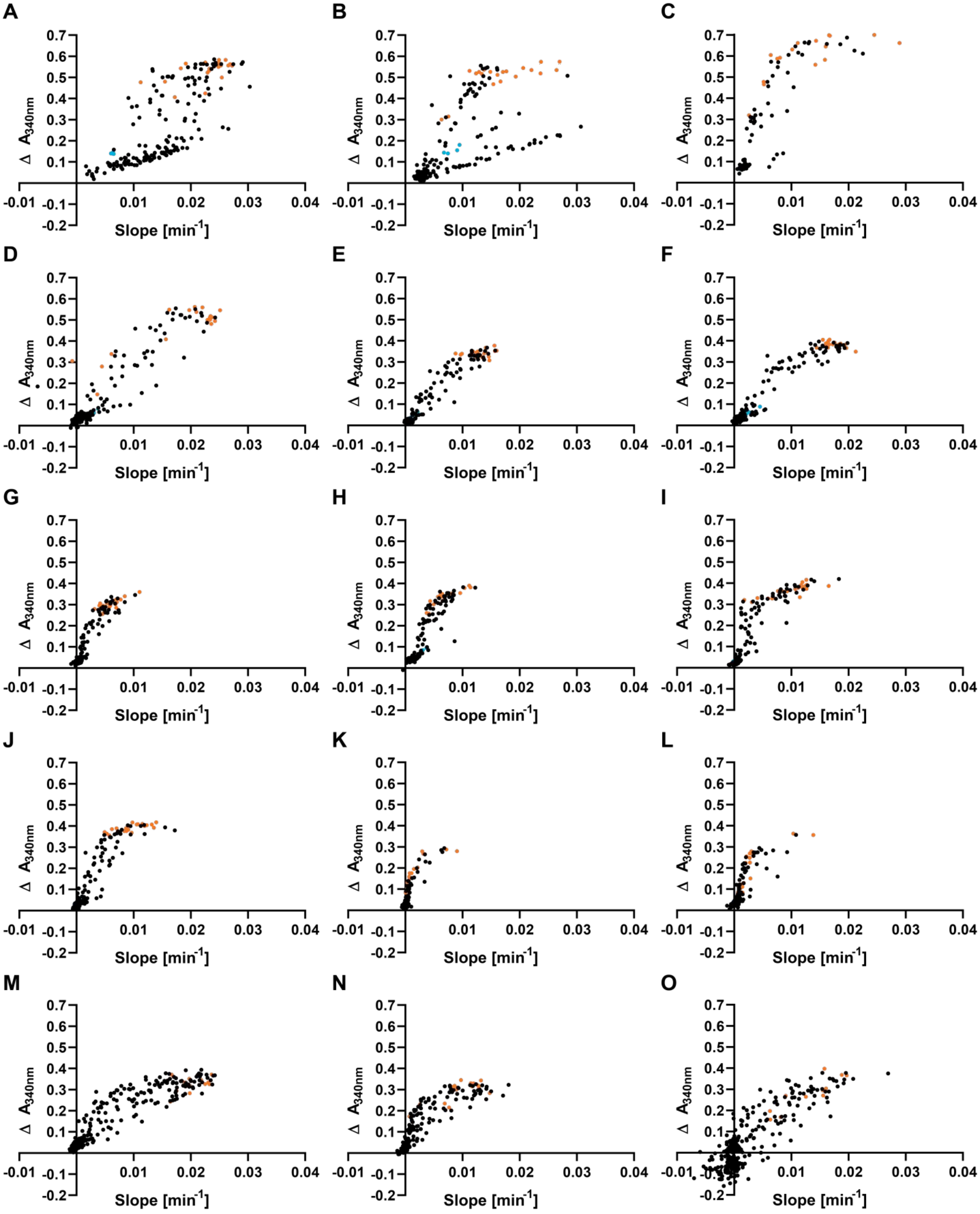

Figure S1 A-O) Lysate-based screen of random mutagenesis library (continued on next page).

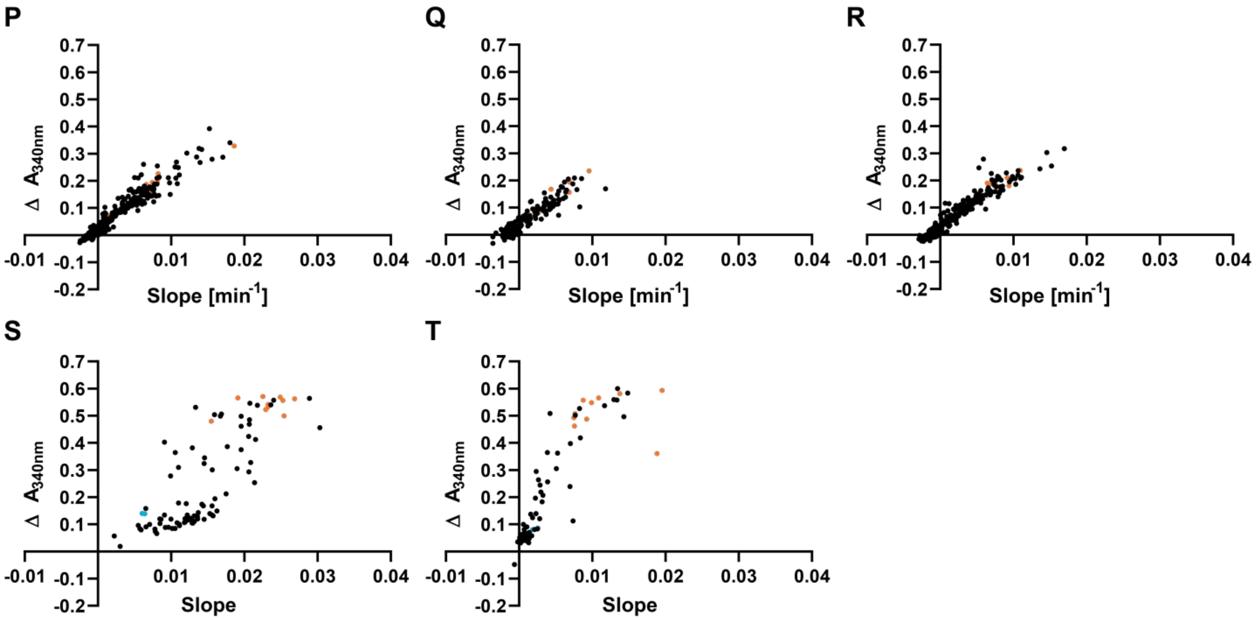

Figure S1 P-T) Lysate-based screen of random mutagenesis library (continued). All graphs show the screening results of the same random mutagenesis library measured in 192-sample batches (per individual 384-well plate) represented in the different graphs. The overall absorbance decrease during the first 10,000 s is plotted on the y-axis and indicative for the ATP per carboxylation ratio. The initial slope of absorbance decrease during the first 500 s of the reaction is plotted on the x-axis and indicative for the carboxylation rate. In total 3840 samples including 3360 randomly mutagenized variants, 400 positive controls with unmutated GCC M5 and 80 negative controls with lysis buffer were measured. Black: randomly mutagenized variants of GCC M5. Orange: positive controls with unmutated GCC M5. Blue: negative controls with lysis buffer (Celllytic B; Sigma Aldrich) instead of cell lysate. A_{340nm}: Absorbance at 340 nm

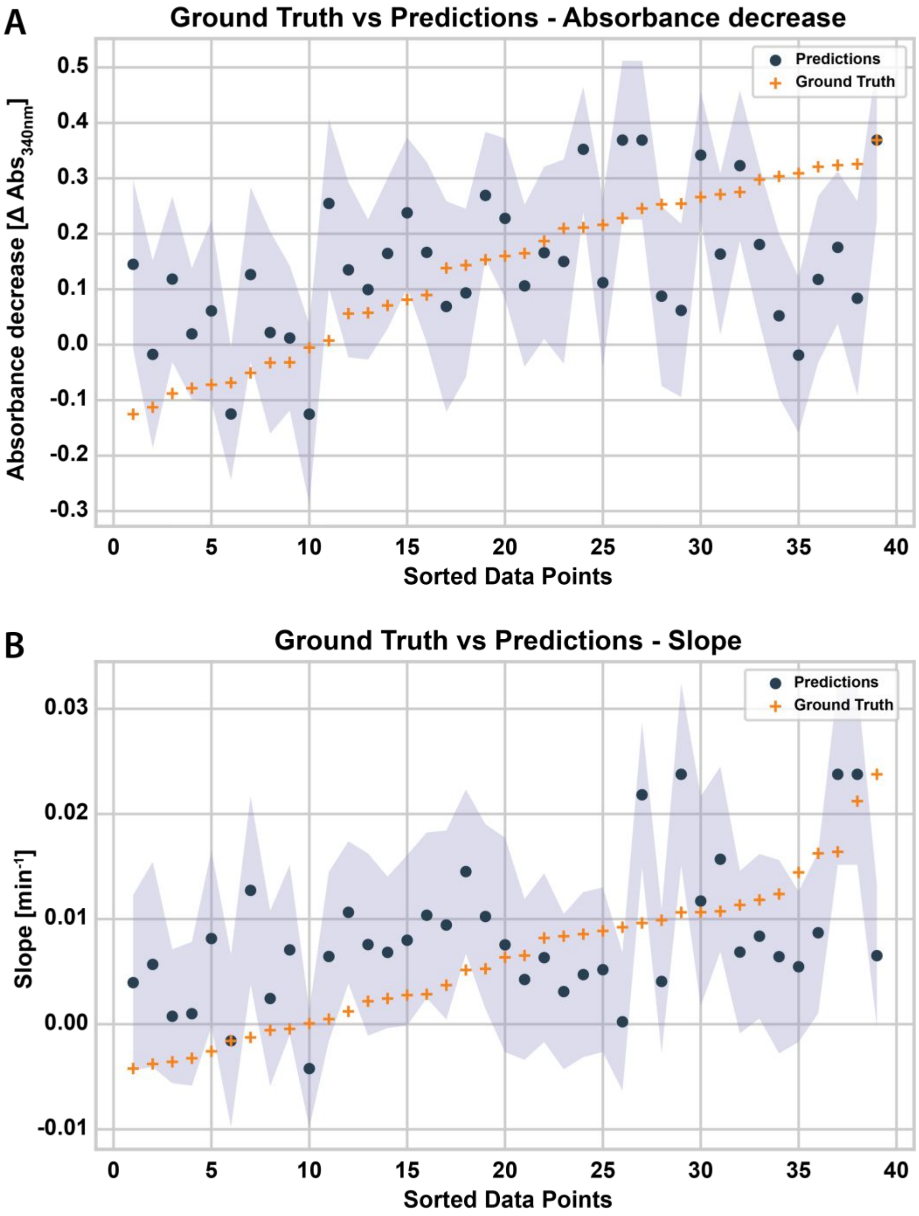

Figure S2. ML-predicted enzyme properties and experimentally measured properties. The ML model was trained on 117 sequences (not shown in the plot) and predicted the 39 remaining sequences to visualize the prediction quality. **A)** Experimentally measured (‘ground truth’, orange crosses) and predicted (blue crosses) absorbance decrease at 340 nm. **B)** Experimentally measured (‘ground truth’, orange crosses) and predicted (blue crosses) data for slope.

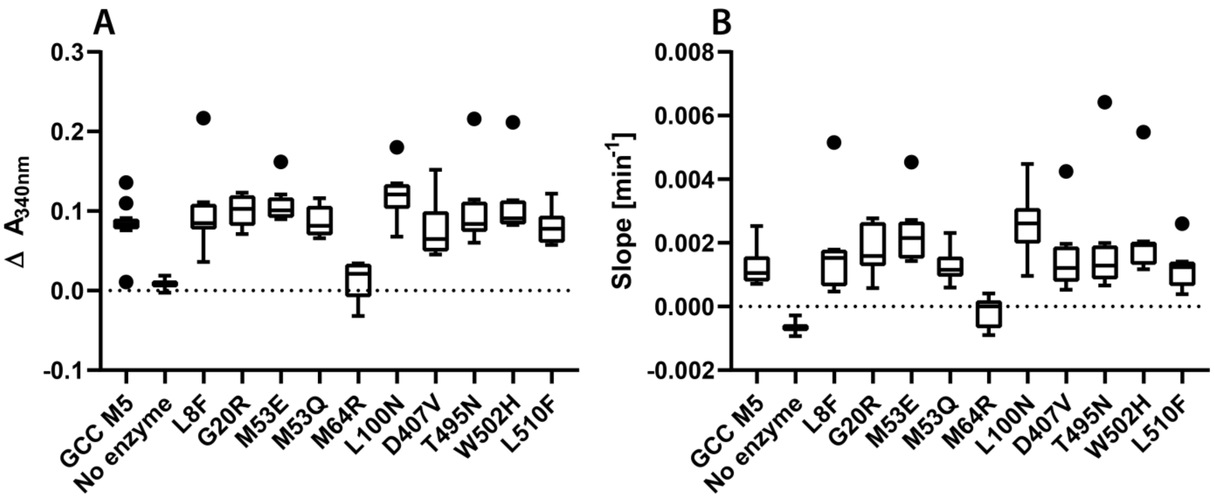

Figure S3. Lysate-based screen of preselected mutant variants. GCC M5 was used as reference and positive control, while a no-enzyme control containing lysis buffer without lysate was used as negative control. **A)** Overall absorbance decrease during the first 10,000 s of the reaction. **B)** Initial slope of absorbance decrease during the first 450 s. Both graphs indicate activity for all mutant variants except for variant M64R. A_{340nm}: Absorbance at 340 nm.

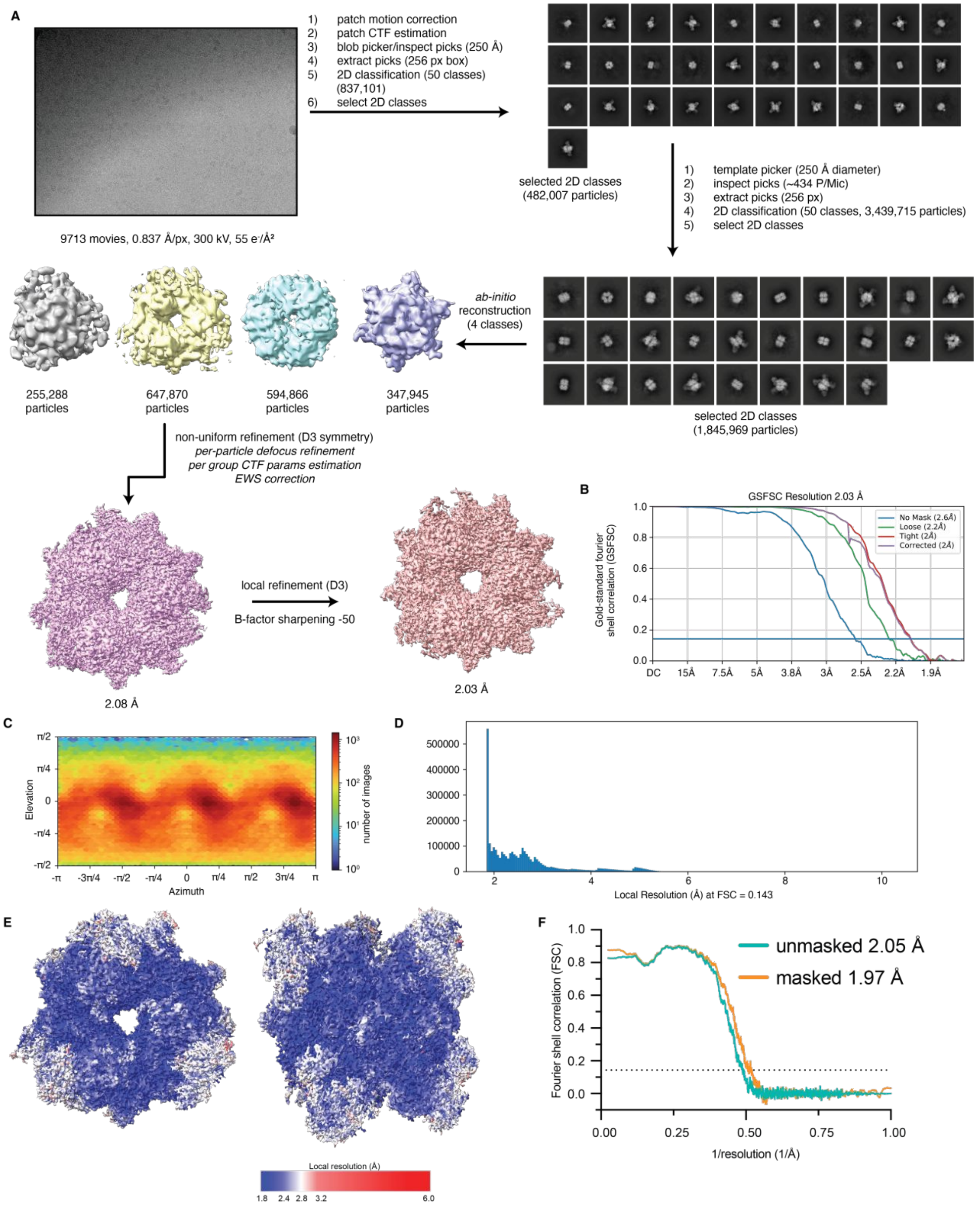

Figure S4. Cryogenic electron microscopy (Cryo-EM) data collection and analysis for GCC M5 G20R. Cryogenic electron microscopy data collection and analysis of GCC M5 G20R. **A)** Schematic processing workflow for the electron map of GCC M5 G20R. Dataset was collected on a Titan Krios G3i electron microscope equipped with a Gatan BioQuantum-K3 imaging filter. **B)** Gold-standard Fourier shell correlation plot from map refinement in CryoSPARC. Resolution determined at Fourier shell correlation (FSC) = 0.143. **C)** Angular particle distribution. **D)** Distribution of local resolution at FSC = 0.143. **E)** Local resolution as calculated by CryoSPARC mapped onto the refined density with different views (top and side view) shown. **F)** Map to atomic model FSC plot with resolution (masked and unmasked) determined at FSC = 0.143.

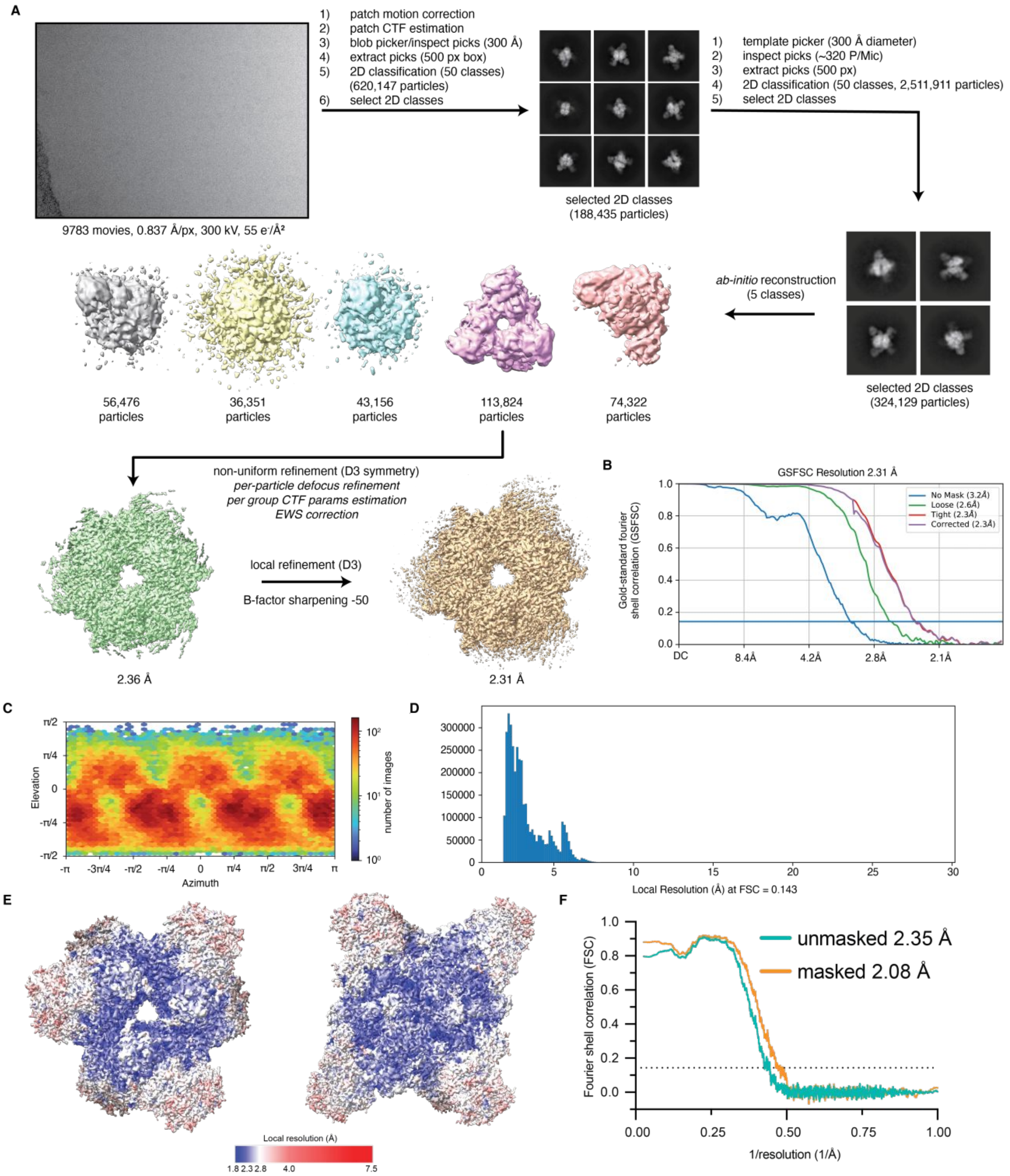

Figure S5. Cryogenic electron microscopy (Cryo-EM) data collection and analysis for GCC M5 L100N. Cryogenic electron microscopy data collection and analysis of GCC M5 L100N. **A)** Schematic processing workflow for the electron map of GCC M5 L100N. Dataset was collected on a Titan Krios G3i electron microscope equipped with a Gatan BioQuantum-K3 imaging filter. **B)** Gold-standard Fourier shell correlation plot from map refinement in CryoSPARC. Resolution determined at Fourier shell correlation (FSC) = 0.143. **C)** Angular particle distribution. **D)** Distribution of local resolution at FSC = 0.143. **E)** Local resolution as calculated by CryoSPARC mapped onto the refined density with different views (top and side view) shown. **F)** Map to atomic model FSC plot with resolution (masked and unmasked) determined at FSC = 0.143.

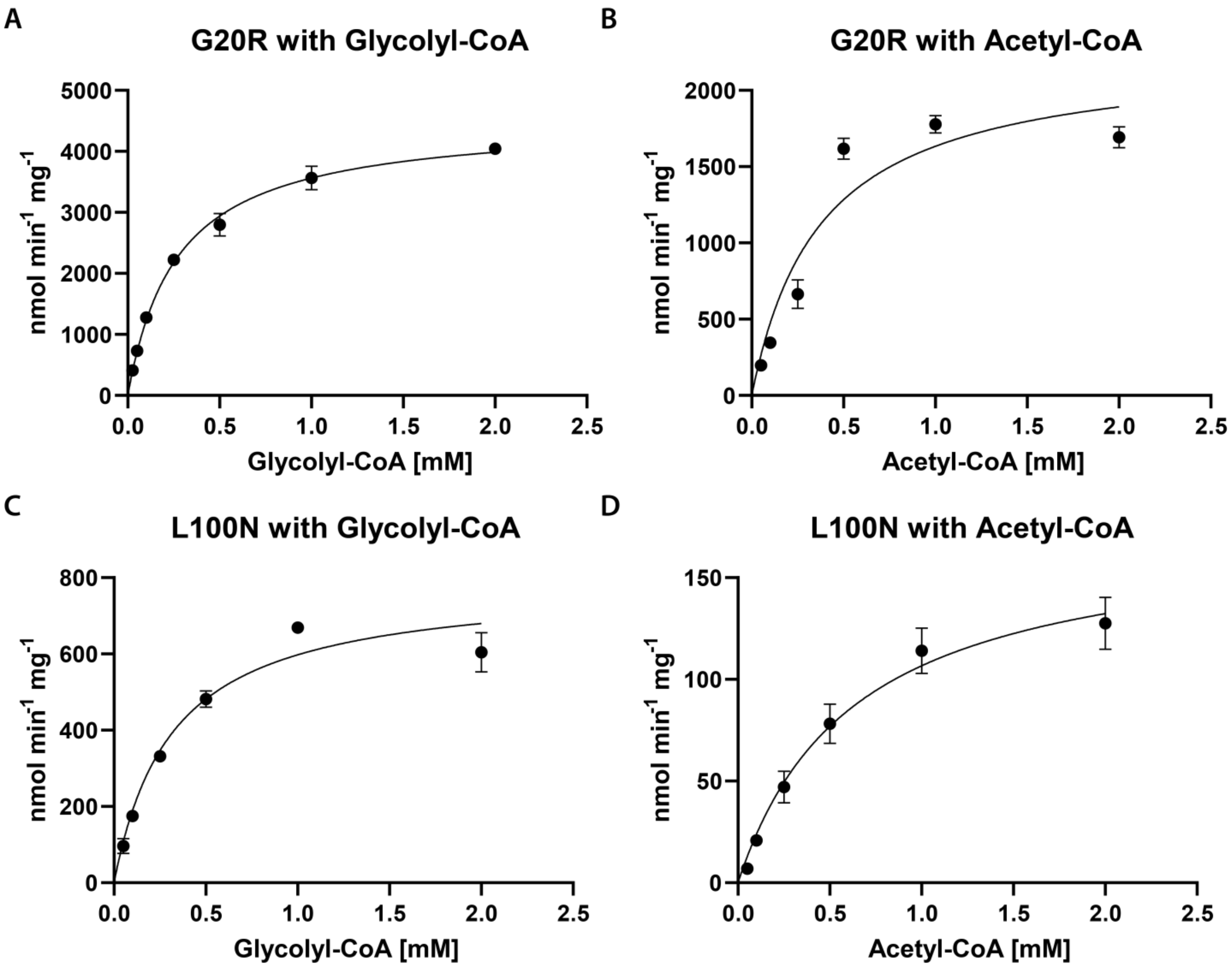

Figure S6. Michaelis-Menten kinetics for GCC M5 G20R and L100N. Michaelis-Menten kinetics were determined via LCMS, each kinetic was measured in triplicates and at least six different substrate concentrations. Quantification of product formation was used to determine enzyme activities. The data were analyzed using nonlinear regression. **A)** GCC M5 G20R with glycolyl-CoA as starting substrate. **B)** GCC M5 G20R with acetyl-CoA as starting substrate. **C)** GCC M5 L100N with glycolyl-CoA as starting substrate. **D)** GCC M5 L100N with acetyl-CoA as starting substrate.

References

[1] Scheffen, M., Marchal, D. G., Beneyton, T., Schuller, S. K., Klose, M., Diehl, C., Lehmann, J., Pfister, P., Carrillo, M., He, H., Aslan, S., Cortina, N. S., Claus, P., Bollschweiler, D., Baret, J.-C., Schuller, J. M., Zarzycki, J., Bar-Even, A., and Erb, T. J. (2021) A new-to-nature carboxylation module to improve natural and synthetic CO_2 fixation, *Nat. Catal. 4*, 105-115.

[2] Kroeger, J. K., Zarzycki, J., and Fuchs, G. (2011) A spectrophotometric assay for measuring acetyl-coenzyme A carboxylase, *Anal. Biochem. 411*, 100-105.
